# Pax3 loss of function delays tumour progression in kRAS-induced zebrafish rhabdomyosarcoma models

**DOI:** 10.1038/s41598-022-21525-5

**Published:** 2022-10-13

**Authors:** A. Kahsay, E. Rodriguez-Marquez, A. López-Pérez, A. Hörnblad, J. von Hofsten

**Affiliations:** 1 Integrative Medical Biology (IMB), Umeå University, Johan Bures Väg 12, 90187 Umeå, Sweden; 2 Umeå Centre for Molecular Medicine (UCMM), Umeå University, Johan Bures Väg 12, 90187 Umeå, Sweden

**Keywords:** Cancer, Cell biology, Genetics

## Abstract

Rhabdomyosarcoma is a soft tissue cancer that arises in skeletal muscle due to mutations in myogenic progenitors that lead to ineffective differentiation and malignant transformation. The transcription factors Pax3 and Pax7 and their downstream target genes are tightly linked with the fusion positive alveolar subtype, whereas the RAS pathway is usually involved in the embryonal, fusion negative variant. Here, we analyse the role of Pax3 in a fusion negative context, by linking alterations in gene expression in *pax3a/pax3b* double mutant zebrafish with tumour progression in kRAS-induced rhabdomyosarcoma tumours. Several genes in the RAS/MAPK signalling pathway were significantly down-regulated in *pax3a/pax3b* double mutant zebrafish. Progression of rhabdomyosarcoma tumours was also delayed in the *pax3a/pax3b* double mutant zebrafish indicating that Pax3 transcription factors have an unappreciated role in mediating malignancy in fusion negative rhabdomyosarcoma.

## Introduction

Rhabdomyosarcoma (RMS) is a rare soft tissue sarcoma with molecular and histological features that resemble undifferentiated skeletal muscle^1^. Based on histological features, RMS tumours can be classified into two major subtypes, embryonal RMS (ERMS) and alveolar RMS (ARMS)^2,3^. ERMS is associated with a variety of mutational events, typically involving components in the Rat sarcoma virus (RAS) pathway^4^, whereas ARMS is characterized by chromosomal translocation resulting in PAX3-FOXO1 or PAX7-FOXO1 fusion proteins, which in humans has led to the replacement of histological annotations ERMS and ARMS with ‘Fusion negative’ and ‘Fusion positive’ RMS^5^. RMS tumours are generally caused by a genetic perturbation associated with muscle progenitor cell proliferation versus cell differentiation. Dysregulation of RAS and its downstream signalling cascade, RAF-MEK-ERK, alters cell cycle progression^6^, transcription of myogenic regulatory factors and ultimately influences myogenic differentiation^7–9^.

During early development, skeletal muscle progenitors are formed from cells within the axial mesoderm. Myogenesis is regulated by myogenic regulatory factors, such as myogenin, MyoD, Myf5, and MRF4^9^. The paired homeobox transcription factors (Pax3 and Pax7) and sine oculis related homeobox (Six1 and Six4) are also important for early lineage specification^10^. Pax3 plays a critical role in cell proliferation, differentiation, and migration during embryonic development of cells^11^, whereas Pax7 is mainly involved in progression of muscle regeneration via muscle stem cells^11^. Myogenic factors regulate normal myogenesis and regenerative processes, but they may also play a role in pathogenic myogenic differentiation, as dysregulation may lead to the development of RMS^12^. In fusion positive RMS, the DNA binding domains of either PAX3 or PAX7 are usually translocated to form a chimeric fusion with the transactive domain of FOXO1, which leads to altered expression of the PAX3/PAX7 downstream targets^13–15^. Less is known on the possible roles of the PAX3/PAX7 genes in fusion negative RMS. Regardless of fusion status, altered levels of transcription factors that regulate the myogenic process are characteristics for RMS cells^16–19^.

Several studies to date have discovered distinct activities of these myogenic transcription factors in the context of normal muscle development and RMS^9–12,20^. However, it remains less clear what factors cause these myogenic regulatory factors to depart from their canonical roles as drivers of muscle differentiation to instead maintain RMS cells as less differentiated muscle progenitors. The gene coding for the transcription factor SIX1, co-expressed with *PAX3* and *PAX7* in myogenic precursors, is highly expressed in human RMS tumours and has been reported to be a regulator of metastasis in mice models for RMS^21^. Being an important developmental factor, the expression of *pax3* gradually decreases as the tissue differentiates. The molecular mechanism of loss of function of *pax3* in RMS formation and progression is not yet clear. However, re-expression of *pax3* in differentiated adult tissues, that require Pax3 function during embryonic development, leads to tumour formation^22,23^.

The zebrafish has emerged as an efficient model for studying molecular and genetic aspects of cancer^24^, including RMS, and several studies have shown similarities between human fusion negative RMS and zebrafish RMS (zRMS)^25^. However, as a consequence of an ancestral genome duplication event in the evolution of teleost fish, the zebrafish genome harbours two separate *pax3* genes, *pax3a* and *pax3b*^26,27^ .The zebrafish *pax3* genes have over-lapping expression patterns and their functions have been shown to be partially redundant during development^28,29^. In zebrafish, fusion negative RMS can be induced by overactivation of the RAS pathway and has been shown to mimic human fusion negative RMS. Similarly, a human PAX3/FOXO1 fusion construct drives fusion positive RMS formation in zebrafish^30^. Given these similarities to human disease, and the lack of knowledge regarding the role of Pax3 in fusion negative RMS, we performed transcriptome analysis of *pax3a/pax3b* double mutant embryos to characterize the downstream targets and possible effectors of Pax3 transcriptional regulation. We also investigated the onset and progression of induced zRMS in *pax3a/pax3b* single and double mutants. This study demonstrates that progression of tumour is delayed in *pax3a*/*pax3b* mutant embryos, and that several genes in the RAS/MAPK signalling pathway are down-regulated in the mutants, linking Pax3 transcriptional regulation to one of the main mediators of malignancy in zRMS. This points to a yet unappreciated role of Pax3 also in fusion negative RMS.

## Materials and methods

### Zebrafish maintenance

All experiments were performed in compliance with national and institutional laws and guidelines and the study is reported in accordance with ARRIVE guidelines. Zebrafish (Danio rerio) used in this study were maintained in compliance with the standard procedures at the Umeå University Zebrafish Facility. All animal experiments were approved by the Regional Ethics Committee at the Court of Appeal of Northern Norrland, Dnr A 6 2020.

### Micro-injection

The *rag2-kRASG12D* plasmid (gift from the Langenau lab, Harvard Medical School, USA) was linearized with *XhoI* (New England Biolabs, MA, USA) as described previously^25^ and purified with NucleoSpin Gel and PCR clean-up mini kit for gel extraction and PCR clean up (Macherey–Nagel GmbH & Co.KG, Germany). 30 ng/µl of the linearized DNA was injected into the 1-cell stage of transgenic *TgBAC(pax3a:EGFP)*^*i150*^^31^ zebrafish crossed with the *pax3a*^*−/−(umu5)*^ and *pax3b*^*−/−(umu6)*^ knock out lines^29^ to generate single and double *pax3a*^*−/−*^ and *pax3b*^*−/−*^ mutants also carrying the *pax3a*:*EGFP* transgenic insertion. Zebrafish larvae were monitored daily and assessed in vivo for tumour onset using dissecting fluorescence microscope. Tumour onset was identified by abnormal accumulation of *pax3a*:*EGFP* in the muscle tissue. zRMS zebrafish larvae were kept separately, examined and photographed every four to five days to evaluate zRMS progression. zRMS tumour area was quantified using ImageJ.

### Semi-quantitative RT- PCR

zRMS were collected at 29 days post fertilization (dpf) to 31 dpf and tumour tissue was extracted using micro incision blade from each fish and used for RNA isolation. Total RNA extraction was performed from each extracted zRMS tumour using RNeasy mini kit (Qiagen) and reverse transcribed to cDNA using SuperScript III reverse transcriptase (Invitrogen), following the manufacturer’s protocol. qRT-PCR was performed on Applied Biosystems VIIA 7 Real-Time PCR System (ThermoFisher Scientific) using FastStart universal SYBR green master mix (Roche). The primer sequences are listed in supplemental Table 1. ß-actin was used as a reference gene.

### Transcriptome sequencing and analysis

Zebrafish embryos from wild type and *pax3a*^*−/−(umu5)*^*; pax3b*^*−/−(umu6)*^ homozygous double mutants were collected at 42 h post fertilization (hpf) for RNA preparation. 9–10 zebrafish larvae of each type were collected and pooled for a sufficient amount of RNA per sample. A total of six samples, two triplicates each, were included in RNA sequencing. Total RNA was extracted using TRizole and treated with TURBO DNase for 15 min at 37 °C to remove genomic DNA. Purity and integrity of the RNA samples was confirmed using Agilent 2100 bioanalyzer.

Poly-A selection libraries were prepared, pooled and sequenced on a 1/4th NovaSeq600 S4 lane, with 2 × 150 bp reads (https://www.scilifelab.se/). Raw reads were aligned to the zebrafish genome (GRCz11, ensemble.org) using STAR (options: -genome Load NoSharedMemory --outSAMtype BAM SortedByCoordinate --seedSearchStartLmax 12 --outFilterScoreMinOverLread 0.3 --alignSJoverhangMin 15 --outFilterMismatchNmax 33 --outFilterMatchNminOverLread 0 --outFilterType BySJout --outSAMunmapped Within --outSAMattributes NH HI AS NM MD --outSAMstrandField intronMotif --quantMode GeneCounts)^32^. Genes with a minimum of 10 reads were kept for further analysis. Normalization and differential expression analyses were performed using DESeq2^33^. Genes were considered differentially expressed with p-value < 0.01 and FDR < 0.01. Clustering of differentially expressed genes was obtained using pheatmap package. Gene ontology, KEGG and Disease ontology enrichment analysis were performed using clusterProfiler software^34^.

### Statistical analysis

Student's t-test and Mann–Whitney U test were used to calculate the significance level on the qRT-PCR, tumour onset and progression analyses. P < 0.05 was considered significant. Error bars indicate mean ± SEM. All the statistical analyses were conducted with GraphPad prism 9 and RStudio.

## Results

### Functional enrichment analysis reveals that the RAS/MAPK signalling pathway is dysregulated following Pax3 loss of function

**Figure 1:**
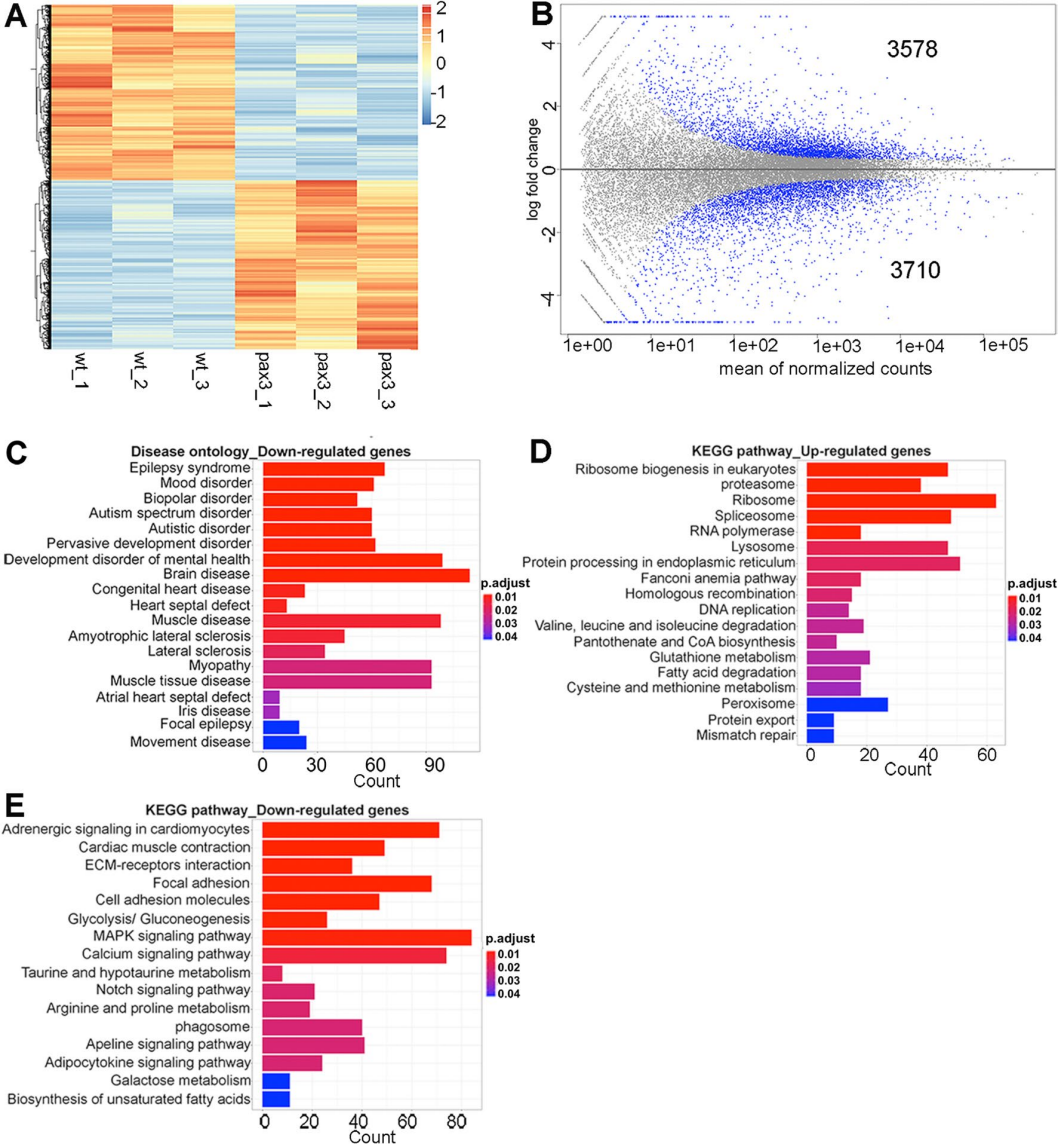
Clustering and pathway analysis of differentially expressed genes (DEGs). **(A)** Heatmap of significant DEGs between wild type and *pax3a*^*−/−*^*;pax3b*^*−/−*^ zebrafish embryos. Regularized log transformed (rlog) count matrix was generated using DESeq2 package. Significant DEGs were used to plot the heatmap of rlog counts. Red and blue represent up- and down-regulated genes, respectively. **(B)** Scatter plot for DEGs in wild type and *pax3a*^*−/−*^*;pax3b*^*−/−*^ showing mean of normalized counts and log2 fold change. All differentially expressed genes (in blue) with adjusted p-value < 0.01. **(C)** Disease ontology analysis of down-regulated genes. **(D,E)** KEGG pathway analysis of up- and down-regulated genes. Bar plots represents the number of DEGs involved in each condition. Adjusted p-value was represented by colour scale, and the statistically significance level decreased from red (highly significant) to blue (relatively lower significant). p-value < 0.01.

To explore downstream transcriptional changes that occur when perturbing *pax3* gene function, RNA-sequencing analysis was performed on whole embryos from *pax3a*^*−/−*^*; pax3b*^*−/−*^ double mutants and wild type zebrafish at 42 hpf. RNA-seq libraries were generated in triplicates for both conditions. Both principal component analysis and hierarchical clustering showed a high overlap between the replicates within each condition while clearly separating the triplicates into two groups (Supplemental Fig. 1A,B). This confirms a high reproducibility in the experiments and suggests that profound transcriptional changes take place in the Pax3 null mutants. Accordingly, differential gene expression analysis demonstrated that 7288 genes were differentially expressed (p < 0.01, FDR < 0.01) between the wild type and mutant embryos, of which 3578 were up-regulated and 3710 down-regulated (Fig. 1A,B, Supplemental Table 2).

To get more insight into the molecular processes and signalling pathways that are altered in the absence of Pax3 function, Gene Ontology (GO), disease ontology, and Kyoto Encyclopedia of Genes and Genomes (KEGG) enrichment analysis were performed separately on genes either up- or down-regulated in the mutants. These analyses revealed that DEGs are enriched for many biological processes and signalling pathways in which Pax3 has an established role, including ncRNA processing, ribosome biogenesis, axonogenesis and neuron development (Supplemental Fig. 1). In order to identify disease conditions in response to loss of Pax3 in zebrafish embryos, we performed disease ontology enrichment analysis and only down-regulated genes in *pax3a*^*−/−*^*;pax3b*^*−/−*^ double mutant zebrafish were implicated in disease. Among the identified diseases we observed examples linked to neurology, such as epilepsy syndrome, autism spectrum disorder, brain diseases, but also diseases related to muscle cell function, like congenital heart disease, muscle tissue diseases and dystrophy (Fig. 1C). Many of the up-regulated genes were involved in ribosome biogenesis and protein processing (Fig. 1D). Intriguingly, among the most highly enriched KEGG terms for the down-regulated genes was the MAPK signalling pathway (Fig. 1E). This is known to be downstream of RAS that is closely connected to zRMS development. Importantly, the MAPK linked group of genes was the largest among the identified KEGG pathways in the analysis. This indicate that Pax3 directly or indirectly contributes to the regulation of this pathway, which may provide a link to the potential role of Pax3 in fusion negative RMS.

**Figure 2:**
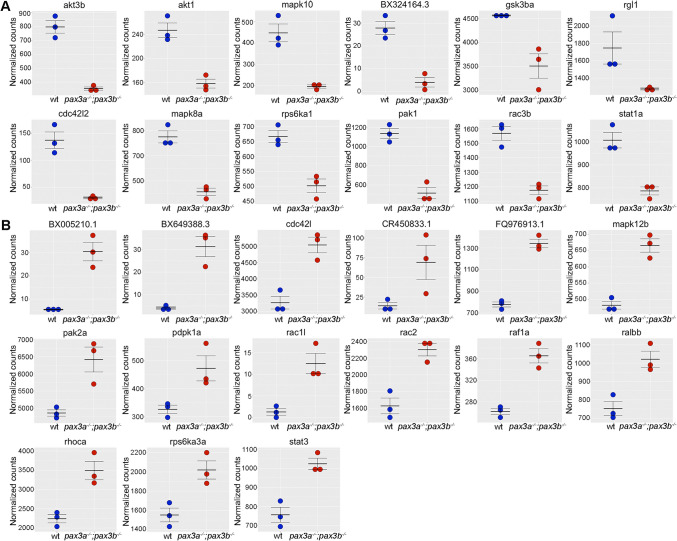
Expression of RAS/MAPK signalling pathway linked genes in wildtype (wt) and *pax3a*^*−/−*^*;pax3b*^*−/−*^ mutant zebrafish embryos: **(A)** Plots of down-regulated genes: *akt1*, *pak1*, *mapk10*, *rps6ka1*, *cdc42l2*, *mapk8a*, *rac3b*, *akt3b*, *BX324164*, *rgl1*, *stat1a* and *gsk3ba.* **(B)** Plots of up-regulated genes: *raf1a, rps6ka3a*, *pak2a*, *FQ976913*, *ralbb*, *rac2*, *CR450833*, *pdpk1a*, *stat3*, *BX005210*, *BX649388*, *mapk12b*, *rhoca*, *cdc42l* and *rac1l.* The normalized counts of each gene in wild type and *pax3a*^*−/−*^*;pax3b*^*−/−*^ mutant is displayed.

To test and validate our findings, we analysed DEGs related to RAS signalling pathway, that have a strong association with rhabdomyosarcoma. 12 genes were down-regulated (Fig. 2A) and 15 genes were up-regulated (Fig. 2B). We further identified common DEGs in RAS and MAPK signalling pathways and found seven down-regulated genes namely *pak1, mapk10, akt3b, rac3b, mapk8a, rps6ka1* and *cdc42l2*.

### In vivo validation of involvement of Pax3 in rhabdomyosarcoma progression

**Figure 3:**
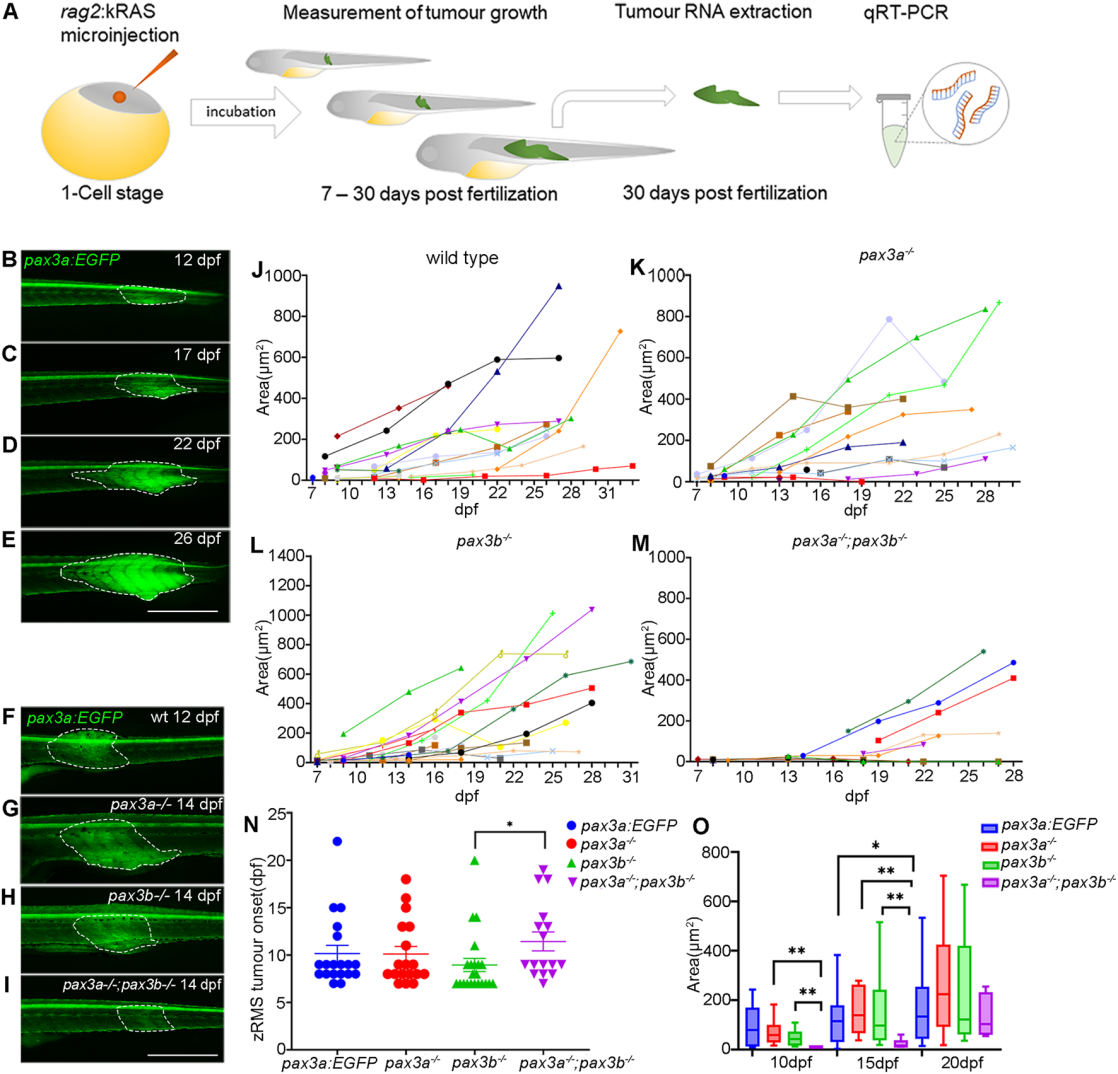
Functional analysis of zRMS progression in wild type and *pax3* mutant zebrafish: **(A)** schematic illustration of generating zRMS, showing microinjection of *rag2:kRASG12D* into one cell stage zebrafish embryo, zRMS detection and monitoring of tumour growth from 7 to 30 dpf, tumour tissue excision and total RNA extraction at 30 dpf for gene expression analysis using qRT-PCR. **(B–E)** zRMS identified in *pax3a:EGFP* zebrafish transgenic line at 12 dpf, 17 dpf, 22 dpf, and 27 dpf. High intensity of GFP expression is observed at the tail area of the zebrafish larvae. Representative image of zRMS tumour progression **(F)** wild type at 12 dpf*,* **(G)** *pax3a*^*−/−*^ at 14 dpf*,* **(H)** *pax3b*^*−/−*^ at 14 dpf and **(I)** *pax3a*^*−/−*^*;pax3b*^*−/−*^ at 14 dpf. Tumour area (μm^2^) of individual zRMS **(J)** wild type fish (n = 24), **(K)** *pax3a*^*−/−*^ (n = 18), **(L)** *pax3b*^*−/−*^ (n = 22), **(M)** *pax3a*^*−/−*^*;pax3b*^*−/−*^ (n = 12). **(N)** zRMS tumour onset is indicated in days post fertilization in wild type fish (n = 20), *pax3a*^*−/−*^ (n = 18)*, pax3b*^*−/−*^ (n = 22) and *pax3a*^*−/−*^*;pax3b*^*−/−*^ (n = 16) zebrafish lines. **(O)** Tumour area (μm^2^) in zebrafish larvae that were injected with *rag2-KRASG12D* in wild type (n = 12), *pax3a*^*−/−*^ (n = 10)*, pax3b*^*−/−*^ (n = 16) and *pax3a*^*−/−*^*;pax3b*^*−/−*^ (n = 6) zebrafish lines at 10 dpf, 15 dpf and 20 dpf. All experiments were conducted in *pax3a:EGFP* background. Tumour areas are indicated with dashed lines. Error bars represent mean ± SEM and significance was calculated using Student t-test and Mann Whitney U test where p < 0.05 was considered significant, *p < 0.05, **p < 0.01, ***p < 0.001. Scale bar: 1 mm.

As the transcriptome analysis show that the MAPK signalling pathway is down-regulated due to loss of *pax3* genes, we intended to determine whether Pax3 is involved in rhabdomyosarcoma formation and progression in zebrafish. We induced zRMS by injecting an oncogene, *rag2-kRASG12D* into one cell-stage of wild type and mutant zebrafish, which lead to spontaneous formation of RMS tumours^25^ (Fig. 3A). zRMS tumours could easily be distinguished from healthy muscle tissue based on the increased *pax3a:EGFP* intensity that characterized the zRMS cells. As the *pax3a:EGFP* construct is randomly integrated in the genome^31^, it is regulated in parallel with the endogenous *pax3a* and its expression remains even after mutation of the endogenous *pax3a* gene. The tumours were followed from onset, when first observed, until around 30 dpf (Fig. 3B–E). The intensity of *pax3a:EGFP* in the zRMS larvae suggests that *pax3a* is highly expressed in zRMS tumours and indicates that Pax3 may play a role in zRMS tumourigenesis. To compare putative roles for the zebrafish *pax3* genes in zRMS tumourigenesis, *pax3a*^+*/−*^*;pax3b*^+*/−*^;*pax3a:EGFP* zebrafish were used to generate *pax3a:EGFP* (used as control), *pax3a*^*−/−*^*;pax3a:EGFP, pax3b*^*−/−*^*;pax3a:EGFP* and *pax3a*^*−/−*^*;pax3b*^*−/−*^*;pax3a:EGFP* zebrafish lines (Fig. 3F–I), and analysed for putative differences in tumour onset, spatial location and growth rate.

The spatial distribution of zRMS tumours did not differ significantly between the examined genotypes, but mainly appeared in either the trunk, the tail or simultaneously in more than one location in the trunk and tail area (Supplemental Fig. 2).

To investigate zRMS growth and progression in single and double *pax3* mutants, we compared zRMS detected in *pax3a:EGFP*, *pax3a*^*−/−*^*;pax3a:EGFP, pax3b*^*−/−*^*;pax3a:EGFP* and *pax3a*^*−/−*^*;pax3b*^*−/−*^*;pax3a:EGFP* zebrafish lines and embryos were observed daily using a fluorescent dissecting scope for putative tumour growth. After tumour detection, carrier zebrafish were kept individually and regularly monitored for tumour progression by measuring the tumour size (Fig. 3J–M). Tumours appeared as early as seven days post fertilization and our results indicated no significant difference between *pax3a*^*−/−*^*;pax3b*^*−/−*^*;pax3a:EGFP* double mutant zebrafish embryos and the wild type (*pax3a:EGFP*) fish (Fig. 3N). Interestingly, we observed that zRMS tumour formation was significantly delayed in *pax3a*^*−/−*^*;pax3b*^*−/−*^*;pax3a:EGFP* double mutant zebrafish compare to the *pax3b*^*−/−*^*;pax3a:EGFP* single mutant zebrafish lines.

In addition to establishing age of the fish for zRMS onset, we compared zRMS tumour areas of all the genotypes at three time points, 10 dpf, 15 dpf and 20 dpf to examine potential significant differences (Fig. 3O). The tumour area of *pax3a*^*−/−*^*;pax3a:EGFP* and *pax3b*^*−/−*^*;pax3a:EGFP* zebrafish lines was significantly higher compared with *pax3a*^*−/−*^*;pax3b*^*−/−*^*;pax3a:EGFP* double mutant zebrafish lines at 10 dpf and 15 dpf. When measured at 15 dpf, the tumour area of *pax3a*^*−/−*^*;pax3b*^*−/−*^*;pax3a:EGFP* double mutant zebrafish line was significantly smaller compared with tumours in all of the other examined genotypes (Fig. 3O). Overall, our in vivo results indicate that loss of function of both Pax3a and Pax3b plays important role in zRMS tumour by delaying growth and progression.

### Expression of DEGs linked to RAS/MAPK signalling in zRMS tumour tissue

**Figure 4:**
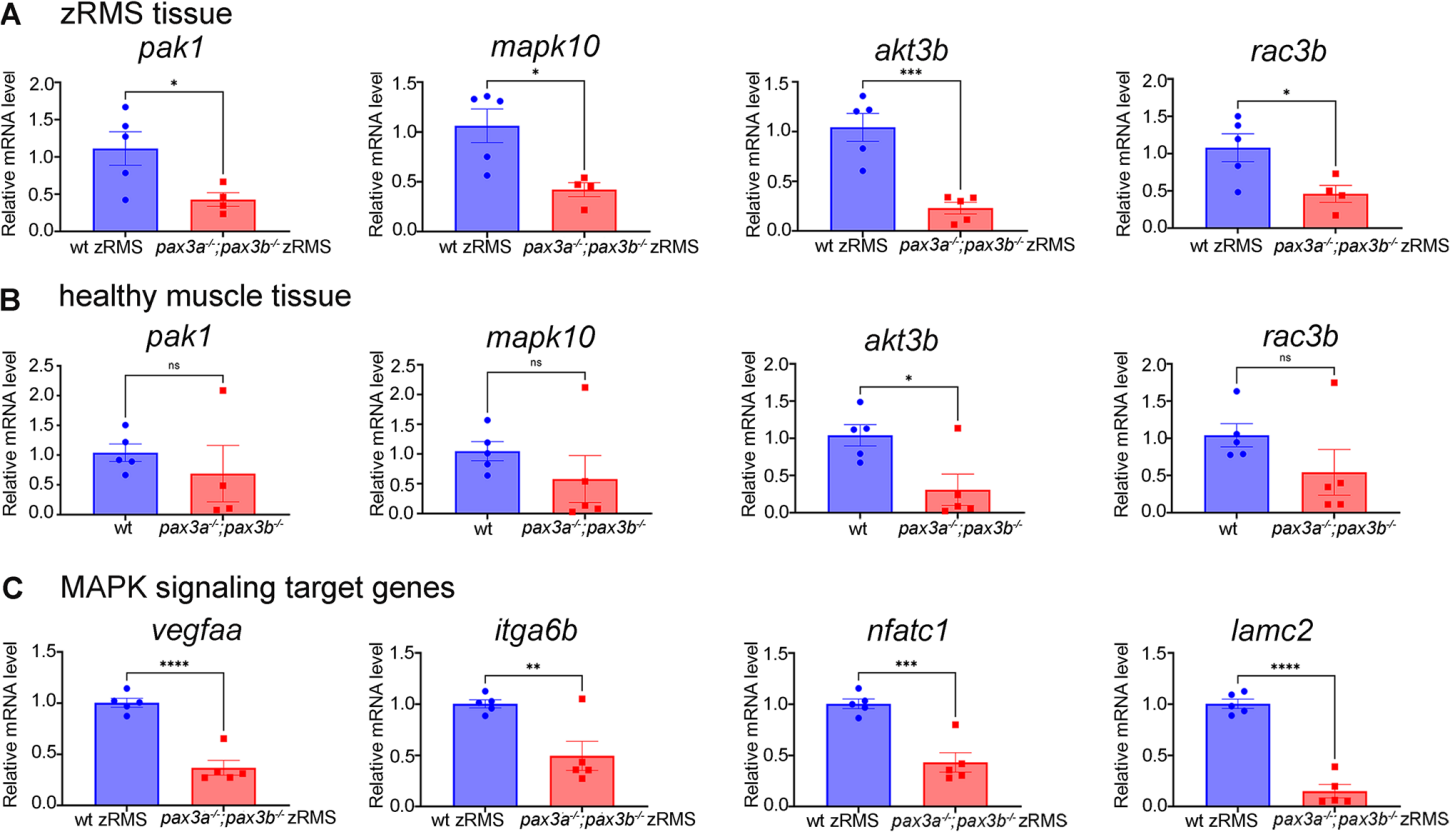
Semi-quantitative RT-PCR for genes linked to RAS/MAPK signalling pathways: **(A)** Relative mRNA expression of *pak1 (*n = 4 *pax3a*^*−/−*^*;pax3b*^*−/−*^ zRMS) , *mapk10*, *akt3b* and *rac3b* in *pax3a*^*−/−*^*;pax3b*^*−/−*^*;pax3a:EGFP* zRMS compared with wild type (*pax3a:EGFP)* zRMS tissue from the similar age*.* **(B)** Relative mRNA expression of *pak1(*n = 4 *pax3a*^*−/−*^*;pax3b*^*−/−*^ ), *mapk10*, *akt3b* and *rac3b* in *pax3a*^*−/−*^*;pax3b*^*−/−*^*;pax3a:EGFP* compared with wild type healthy muscle tissue from the similar age group. **(C)** Relative mRNA expression of *vegfaa, itga6b, nfatc1 and lamc2* in *pax3a*^*−/−*^*;pax3b*^*−/−*^*;pax3a:EGFP* zRMS compared with wild type (*pax3a:EGFP)* zRMS tissue*.* n = 5 in all samples unless otherwise stated. Error bars indicate mean ± SEM and significance was calculated using student t-test where p < 0.05 was considered significant, *p < 0.05, **p < 0.01, ***p < 0.001, ****p < 0.0001.

To investigate whether Pax3 is indeed a regulator of the RAS/MAPK pathway in the context of fusion negative RMS, we selected the four genes (*pak1, mapk10, akt3b* and *rac3b*) common to RAS and MAPK signalling among the genes that were down-regulated in our RNA-seq analysis (Fig. 2A), and performed qRT-PCR analysis on cDNA prepared from healthy muscle and zRMS samples, from wild type, single and double mutants (Fig. 4; Supplemental Fig. 3).

Tumours were harvested at 29–31 dpf (Fig. 3A). As a comparison, we used healthy muscle tissue of zebrafish that were raised to the same age group as the zRMS bearing fish. Interestingly, *pak1, mapk10, akt3b* and *rac3b* were down-regulated in *pax3a*^*−/−*^*;pax3b*^*−/−*^*;pax3a:EGFP* zRMS when compared to wild types zRMS (Fig. 4A). The down-regulated gene expression was observed both in tumours and in healthy tissue, but only *akt3b* was significantly lower in *pax3a*^*−/−*^*;pax3b*^*−/−*^ mutants in healthy muscle tissue compared to wild type (Fig. 4B). The differences between wild types and *pax3a*^*−/−*^*;pax3b*^*−/−*^ mutants were however more pronounced when analysing tumour RNA, where all four examined genes were significantly lower compared to wild types. Also, when we examined the expression of the four genes in zRMS tumours in single mutants (*pax3a*^*−/−*^ and *pax3b*^*−/−*^) respectively, no differences were observed (Supplemental Fig. 3), leading to the conclusion that Pax3a and Pax3b may have compensatory functions in the regulation of these genes. Altered MAPK signalling has been shown to affect gene expression in numerous genes^35,36^. Therefore, we analysed the relative expression level of four of these genes (*vegfaa*, *itga6b*, *nfatc1* and *lamc2*), in zRMS from wildtype and *pax3a*^*−/−*^*;pax3b*^*−/−*^ mutants and found that they were expressed significantly lower in double mutant zRMS than in wildtype zRMS (Fig. 4C), in line with the altered MAPK signalling observed. We confirmed that these genes also were expressed significantly lower in *pax3a*^*−/−*^*;pax3b*^*−/−*^ mutant embryos, compared to wildtypes in the transcriptome analysis (Supplemental Fig. 4).

## Discussion

Developing complementary animal models for RMS will help to understand the molecular mechanism of the disease and to identify new therapeutic directions. Zebrafish rhabdomyosarcoma models include a high degree of conservation between zebrafish and human genomes and molecular signalling networks. The zebrafish genome can be edited to assay functions of potentially important RMS oncogenes and genes involved in the progression of the disease. In this study, we induced zRMS by overexpressing the *rag2:kRASG12D* human oncogene and utilized lineage tracing of the zRMS cell of origin to quantify tumour progression in vivo in genetically modified zebrafish, where we identified new roles for the zebrafish transcription factor Pax3a and its paralogue Pax3b in fusion negative RMS.

In fusion positive RMS, the PAX3/FOXO1 fusion protein contributes to tumourigenesis by binding to PAX3 binding sites in target genes, and causing an aberrant change in expression of these downstream targets that results in alveolar subtype^37–40^. However, the embryonal subtype usually involves alterations in the RAS signalling pathway. Although it is known that *PAX3* and *PAX7* genes are associated with the fusion-positive alveolar subtype, further work is needed to clarify the role of *PAX3* in a fusion-negative context. It has been shown, both in humans and in animal models such as zebrafish, that Pax3 is expressed in migrating myoblasts during normal myogenesis and it is believed to inhibit their differentiation until they reach their destination^41–44^. However, the potential role of Pax3 in fusion negative RMS formation and progression remains unclear.

In this study, we presented transcriptional profile of *pax3a*^*−/−*^*;pax3b*^*−/−*^ double mutant zebrafish. Interestingly, the functional enrichment analysis singled out genes linked to RAS/MAPK signalling pathways, where the *pak1*, *mapk10*, *akt3b* and *rac3b* were highlighted due to their association with fusion negative RMS progression. The MAPK pathway encompasses different signalling pathways of which RAS-RAF-MAPK/ERK kinase and the extracellular signal-regulated kinase 1 and 2 (ERK1/2) are one of the most dysregulated in human cancer^45^. This pathway regulates multiple critical cellular functions including proliferation, growth and apoptosis^46^. RAS proteins function as binary molecular switches that control intracellular signalling pathways involved in fundamental cellular processes such as cell polarity, proliferation, differentiation, adhesion, migration and apoptosis^47,48^. RAS and RAS related proteins are often dysregulated in cancers by activating mutation of RAS isoforms or its effectors in nearly one third of human cancers^49^.

The p21-activated PAK1 is one of the critical effectors of the small GTPase Rac1 and Cdc42^50^. Previous studies demonstrated that overexpression of *pak1* in cancer cells increases cell migration potential^51^. Comparably, our RNA-seq and qRT-PCR results showed that *pak1* was down-regulated in *pax3a*^*−/−*^*;pax3b*^*−/−*^ double mutants, in particular in the slow progressing tumours, which makes *pak1* a plausible driver of fusion negative RMS progression in our model. It has been shown that inhibition of MAPK signalling pathway reduces the size and tumourigenicity of stem-like cell populations^52^. In the same study, the potential advantage of using MAPK signalling pathway inhibitors to target embryonal RMS was suggested. Similarly, both our transcriptome and qRT-PCR analyses indicated that *pak1, mapk10*, *akt3b* and *rac3b* were significantly reduced in *pax3a*^*−/−*^*;pax3b*^*−/−*^ double mutants and thereby putatively contributing to a delay in zRMS development by inhibition of MAPK signalling activity. The expression of *pak1*, *mapk10*, *akt3b* and *rac3b* was also slightly lower in healthy muscle tissue from *pax3a*^*−/−*^*;pax3b*^*−/−*^ double mutants in comparison with wildtype controls, but only *akt3b* was significantly reduced (Fig. 4B). The reduction of *akt3b* was more pronounced when comparing tumour tissue (Fig. 4A). In addition to the role of MAPK signalling in regulating proliferation, Akt3 has previously been linked to regulation of vascularization in tumours, by activating the vascular endothelial growth factor (VEGF)^53^. To examine putative downstream effects of altered MAPK signalling, we analysed four known downstream target genes (*vegfaa*, *itga6b*, *nfatc1* and *lamc2*), that previously have been shown to be down-regulated when MAPK is inhibited^36,54^. As predicted, all these genes were significantly reduced in the zRMS from *pax3a*^*−/−*^*;pax3b*^*−/−*^ double mutants compared to wildtype zRMS (Fig. 4C), in agreement with the reduced expression of the genes higher up in the MAPK signalling hierarchy.

Interestingly, our results showed a significant delay in tumour growth in *pax3a*^*−/−*^*;pax3b*^*−/−*^ mutants at 10 and 15 dpf, suggesting an important role of *pax3* genes in tumour progression. The delay in *pax3a*^*−/−*^*;pax3b*^*−/−*^ mutants was less prominent after 20 dpf, likely due to an increased variability in tumour size in all genotypes over time. Due to a putative genetic redundancy in zebrafish, the delayed tumour progression was not observed in single *pax3a*^*−/−*^ or *pax3b*^*−/−*^ mutants. We did not observe any delay in tumour initiation in any of the mutant genotypes compared with wildtypes, but a significant delay was found in *pax3a*^*−/−*^*;pax3b*^*−/−*^ when compared with *pax3b*^*−/−*^ mutants indicating loss of both *pax3* genes affects zRMS biology and behaviour to a higher extent than single mutations. This was also confirmed by the qRT-PCR analyses (Fig. 4; Supplemental Fig. 3), which argues for a redundance between the *pax3a* and *pax3b* genes. A recent study reported that *six1b* knockout alone, without mutation of *six1a*, inhibits zebrafish RMS growth and progression^55^, which shows that redundancy between paralogues not always is the case in this context. Mutation of *six1b* resulted in a change in Myod cis-element occupancy and thereby altered myogenic progression^55^. Hence, even though Pax3 and Six1 are linked to similar genetic programmes in myogenesis and that mutation of Pax3 and Six1 both lead to inhibition of zRMS in zebrafish, the downstream effects of the mutations appear to differ. However, similar to Six1, Pax3 acts upstream of Myf5 in myogenesis^56^ and Myf5 can impart tumour propagating potential to zRMS cells in the zebrafish model^57^. In addition, Myf5 or Myod knockdown suppress RMS proliferation *in vitro*^57^. The Pax3/Foxo1 gene product has also been shown to dirceclty regulate the *myod* and *myog* gene promoters in fusion positive RMS^13^. The myogenic factors *myod* and *myf5* were however not significantly altered in our transcriptome analysis, possibly due to the analysis being performed on whole embryos rather than muscle tissue, but would unlikely be the only cause of delayed tumour progression in the *pax3a*^*−/−*^*;pax3b*^*−/−*^ mutants. In addition to *myod*, previous studies in myoblast and rhabdomyosarcoma cells have shown that *pax3* also is involved in supressing apoptosis and regulation of tumour suppressor genes^58–60^. Together with our data, this indicates that there are parallel pathways involved in RMS progression among the downstream targets of *pax3*, which include MAPK signalling*.*

In conclusion, Pax3a and Pax3b redundantly regulate genes linked to RAS/MAPK signalling both in the context of development and in zRMS progression. This provides new insights into investigating a molecular mechanism of fusion negative RMS progression and a new role for Pax3 in this context.

## Supplementary Information

Supplementary Information.

Supplementary Table 2.

## Data Availability

All generated datasets for RNA-seq are available on the Gene Expression Omnibus repository under the accession number GSE208266.
