# Supplementary material for: Pax3 loss of function delays tumour progression in kRAS-induced zebrafish rhabdomyosarcoma models
Source: Sci Rep. 2022 Oct 13;12:17149. doi: 10.1038/s41598-022-21525-5 (PMC9561152; doi:10.1038/s41598-022-21525-5)
Supplement: Supplementary file 1 — Supplementary Information. [file 41598_2022_21525_MOESM1_ESM.pdf]

SUPPLEMENT DATA

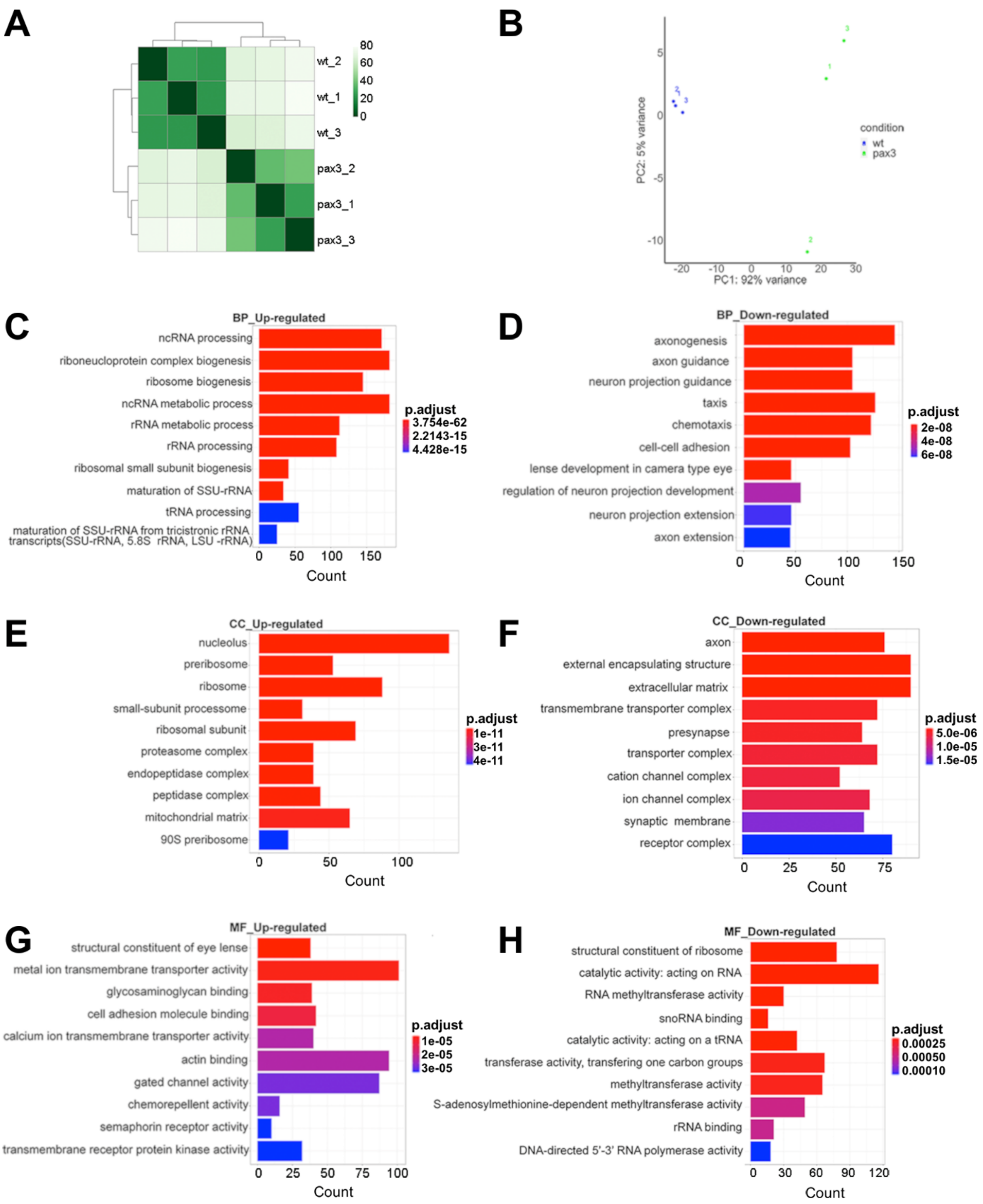

Supplemental Figure 1: Clustering and GO enrichment analysis of DEGs: (A & B) Heatmap and PCA analysis of RNA seq data of *pax3a*$^{-/-}$;*pax3b*$^{-/-}$ mutants and wild type. (C-H) Top 10 GO enrichment analysis of DEGs (up- and down-regulated genes) in zebrafish embryos based on (C & D) biological

process (BP), (E & F) cellular component (CC) and (G & H) molecular function (MF). Adjusted p-value was represented by colour scale, and the statistically significance level decreased from red (highly significant) to blue (relatively lower significant).

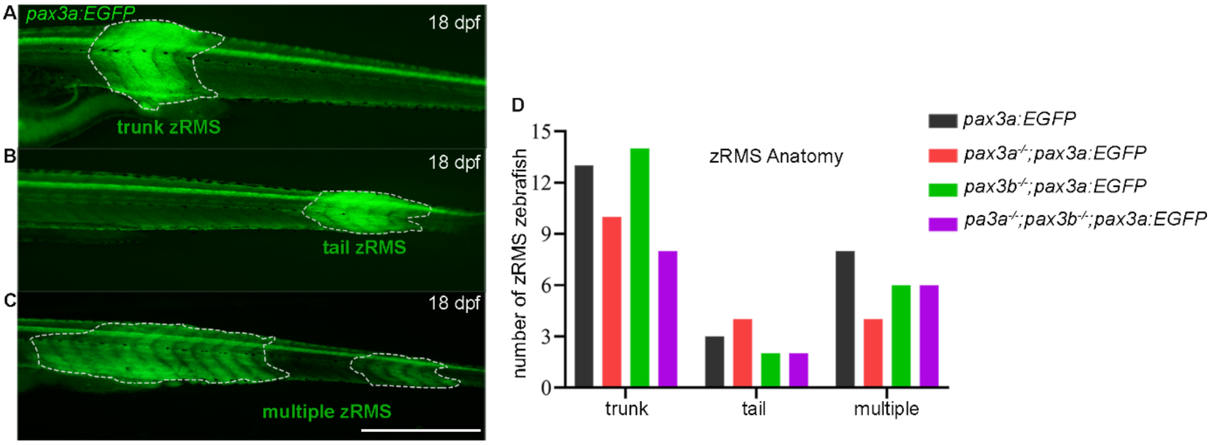

Supplemental Figure 2: Anatomy of zRMS in zebrafish**:** Anatomical representation of zRMS generated in wild type (*pax3a:EGFP)* zebrafish transgenic line indicating different zRMS tumour locations. zRMS in the (A) trunk area, (B) tail area or (C) multiple areas of wild type fish imaged at 18 dpf. (D) Number of zRMS based on spatial location across genotypes. Tumour areas are indicated with dashed lines. Scale bar: 1mm.

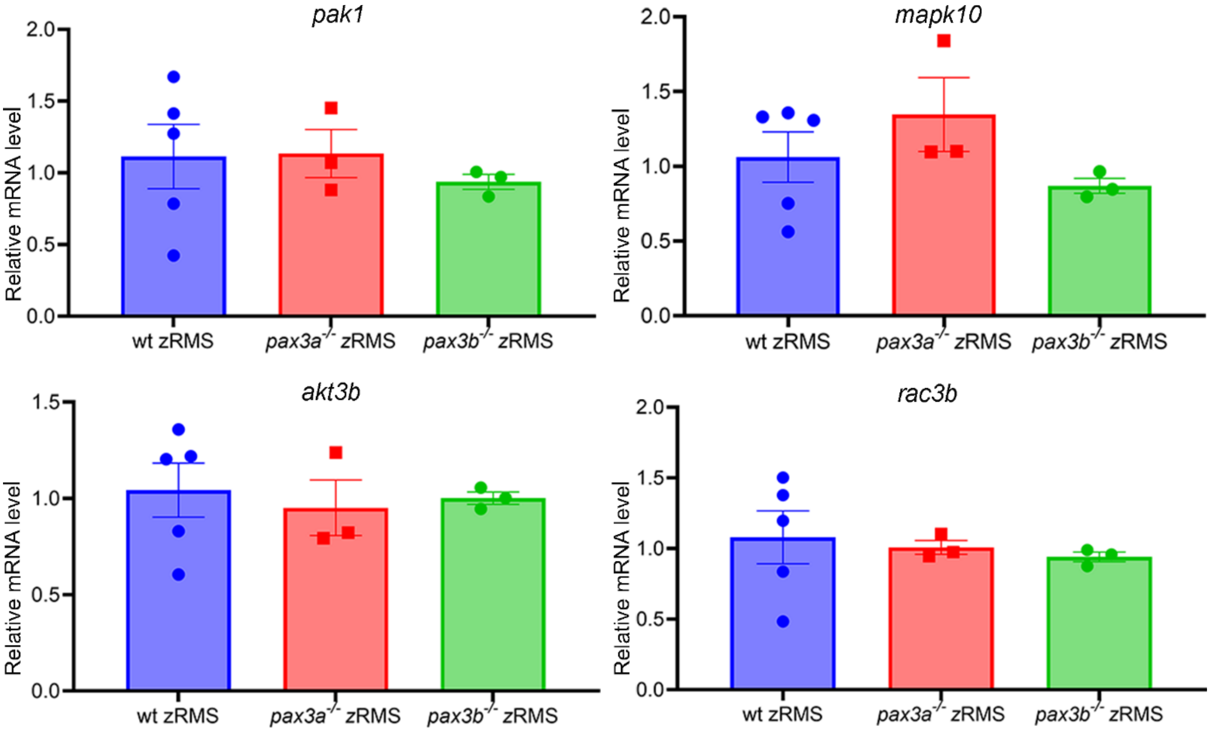

Supplemental Figure 3: qRT-PCR *of pax3a*$^{-/-}$ *and pax3b*$^{-/-}$ *zRMS* in comparison with wild type zRMS : Relative mRNA expression of *pak1*, *mapk10*, *akt3b* and *rac3b* in *pax3a*$^{-/-}$ zRMS and *pax3b*$^{-/-}$ zRMS compared with wild type zRMS from the same age group (n=5 wt zRMS, n= 3 *pax3a*$^{-/-}$ zRMS, n= 3 *pax3b*$^{-/-}$ zRMS) in all samples. Error bars indicate mean$\pm$SEM and significance was calculated using student t-test where $p<0.05$ was considered significant, *$p<0.05$.

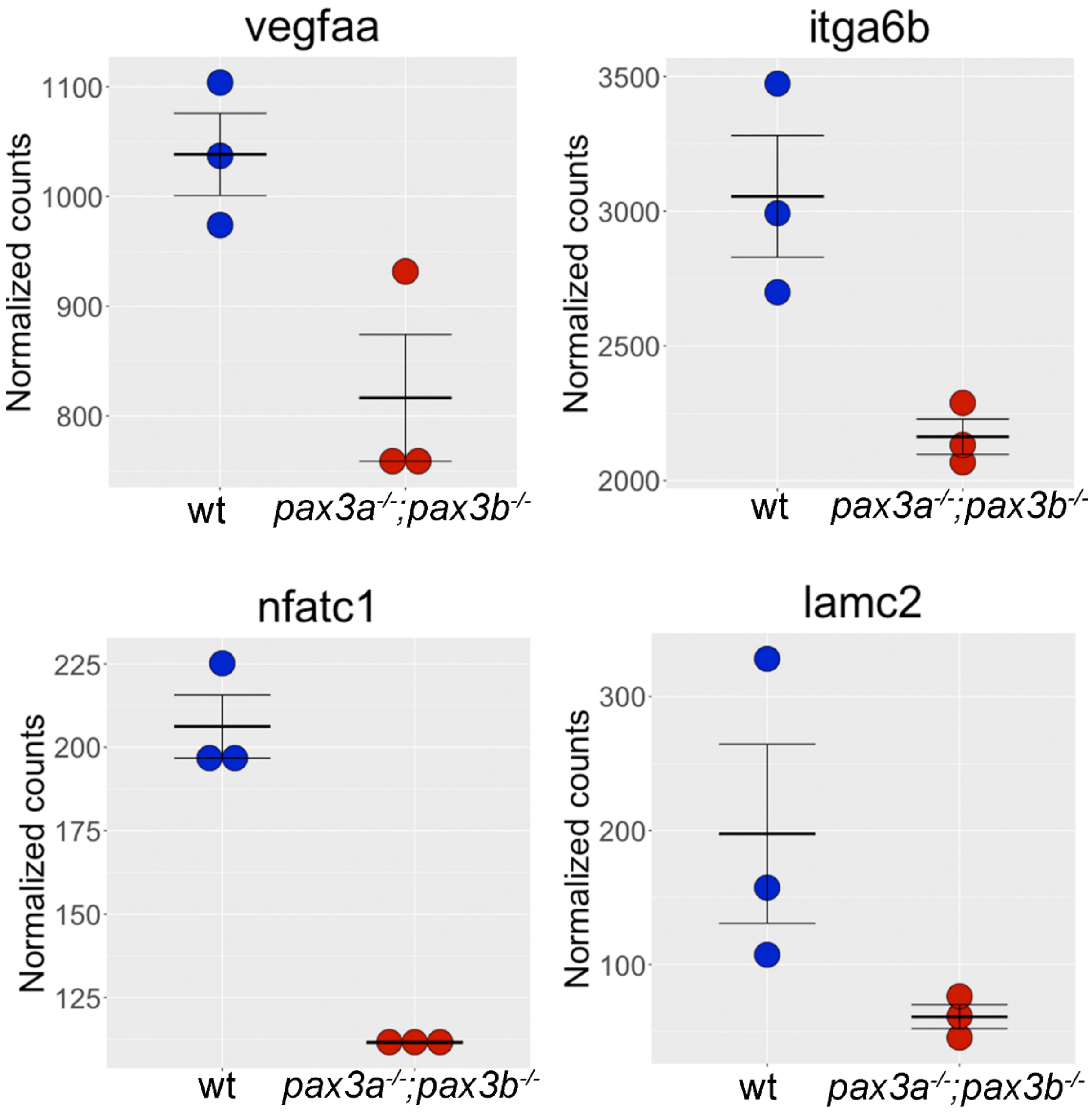

Supplemental Figure 4: Expression of RAS/MAPK signalling pathway downstream target genes in wildtype (wt) and *pax3a*$^{-/-}$;*pax3b*$^{-/-}$ mutant zebrafish embryos: Plots of down-regulated genes; *vegfaa, itga6b, nfatc1* and *lamc2.* The normalized counts of each gene in wild type and *pax3a*$^{-/-}$;*pax3b*$^{-/-}$ mutant is displayed ($p<0.05$).

Supplemental Table 1: list of primer sequences for qRT-PCR

Gene	Primes	Accession number
pak1	F: 5'-CATCGAGATGATCGAGGGC-3'<br><br>R: 5'-GAAAATCCCGAAATACAGCAG-3'	XM_021467598.1
mapk10	F: 5'-TCCCTCAGGTACTCTCTGTGC-3'<br><br>R: 5'-GTCGGTGGACATGGAGGAGA-3'	XM_009295167.3
akt3b	F: 5'-TTCTCTGTCGCCAAGTGTCAGCT-3'<br><br>R: 5'-ACCCACTCGTCCCTCTCCTCT-3'	XM_001923419.7
rac3b	F: 5'-ATTTACGCCGGTGAAGCGAA-3'<br><br>R: 5'-GGACGGAGGCGGTCATAATC-3'	NM_001320406.1
vegfaa	F: 5'- TGTAATGATGAGGCGCTCGAA -3'<br><br>R: 5'- AGGCTCACAGTGGTTTTCTT -3'	XM_009292018.3
Itga6b	F: 5'- ACATTCACAACCCCACCAGAA -3'<br><br>R: 5'- TACTCGCTGGCATGACTGAC -3'	NM_001013448.1
nfatc1	F: 5'- TGCGTTTTGGAAAAGTGCGT -3'<br><br>R: 5'- ATTGTGGTACTGCGAGGGTG -3'	NM_001045159.1
lamc2	F: 5'- AAGAAAGCCAGGAACGCAGT -3'<br><br>R: 5'- CACACGTCTGCGGCTTTTAG -3'	XM_003197884.5
B-actin	F: 5'- GCCTTCCTTCCTGGGTATGG -3'<br><br>R: 5'- CCAAGATGGAGCCACCGAT -3'	NM_001101

Supplemental Table 2: Differentially expressed genes between wild type and pax3 mutants, KEGG and GO term enrichment of up-and down-regulated genes, and Disease ontology (DO) of down-regulated genes (See supplementary excel file "Supplemental_table_2")
